# Performance of the Large Language Models on the Chinese National Nurse Licensure Examination: Cross-Sectional Evaluation Study

**DOI:** 10.2196/78279

**Published:** 2025-11-03

**Authors:** Longhui Xu, Xiao Cong, Renxiu Wang, Na Li, Xinru Liu, Ronghui Wang, Cuiping Xu

**Affiliations:** 1 School of Nursing, Shandong University of Traditional Chinese Medicine, Jinan, China; 2 Department of Nursing, Shandong Provincial QianFoShan Hospital, No.16766 Jingshi Road, Jinan, 250014, China, 86 13791126826

**Keywords:** large language models, artificial intelligence, nursing education, accuracy, reliability, confidence, robustness

## Abstract

**Background:**

Large language models (LLMs) are increasingly explored in nursing education, but their capabilities in specialized, high-stakes, culturally specific examinations, such as the Chinese National Nurse Licensure Examination (CNNLE), remain underevaluated, making rigorous evaluation crucial before their adoption in nursing training and practice.

**Objective:**

This study aimed to evaluate the performance, accuracy, repeatability, confidence, and robustness of 4 LLMs on the CNNLE.

**Methods:**

Four LLMs (Sider Fusion [Vidline Inc], GPT-4o [OpenAI], Gemini 2.0 Pro [Google DeepMind], and DeepSeek V3) were tested on 237 multiple-choice questions from the 2024 CNNLE. Accuracy and repeatability were assessed using 2 prompting strategies. Confidence was evaluated via self-ratings (1‐10 scale) and robustness via repeated adversarial prompting.

**Results:**

DeepSeek V3 and Gemini 2.0 Pro demonstrated significantly higher overall accuracy (ranging from 199/237 to 209/237; >83%) compared to GPT-4o and Sider Fusion (ranging from 151/237 to 166/237; <71%). However, all LLMs showed suboptimal repeatability (highest at 206/237; <87% consistency). Critically, poor confidence calibration was evident; most models showed high confidence often mismatching actual accuracy (Sider Fusion: *P*=.01; GPT-4o: *P*=.03; and Gemini 2.0 Pro: *P*=.049). A stability-flexibility trade-off paradox was also observed.

**Conclusions:**

While some LLMs show promising accuracy on the CNNLE, fundamental reliability limitations (poor confidence calibration and inconsistent repeatability) hinder safe application in nursing education and practice. Future LLM development must prioritize trustworthiness and calibrated reliability over surface accuracy.

## Introduction

The Chinese National Nurse Licensure Examination (CNNLE) is a national professional qualification certification that comprehensively evaluates whether nursing students possess the requisite professional knowledge, clinical skills, and professionalism necessary to independently provide basic nursing services [1]. The examination’s difficulty is comparable to the competency level expected of a nurse with 1 year of practice following the completion of secondary nursing education. Only nursing students who successfully pass this examination are eligible to receive the basic nursing qualification certificate and apply for registration as a nurse. However, within traditional preparation frameworks, nursing students often encounter multiple challenges, such as inefficient revision scheduling, unclear learning pathways, and a lack of personalized guidance [2]. Fortunately, the rapid advancement of artificial intelligence (AI) technology presents new opportunities for nursing education. Personalized learning methodologies driven by big data are anticipated to effectively address these challenges, thereby optimizing the learning experience and enhancing students’ preparation outcomes [3-5].

Large language models (LLMs), intelligent algorithms trained on extensive textual datasets, are rapidly emerging as pivotal drivers of pedagogical innovation in clinical training within nursing education, owing to their remarkable capabilities in personalized learning, clinical scenario simulation, and workload reduction [6-8]. While LLMs have demonstrated promising potential within nursing education, their inherent uncertainty in professional contexts demanding highly complex clinical reasoning and critical thinking remains a frontier of ongoing scholarly investigation [9-11]. A relevant study by Kaneda et al [12] evaluated the performance of GPT-4 and GPT-3.5 on the 112th Japan National Nursing Examination, revealing an overall accuracy of 79.75% (189/237) for GPT-4 and only 59.92% (142/237) for GPT-3.5. This disparity may be attributed to the models’ capacity to interpret shared cultural norms and anticipate unstated intentions. Building on Kaneda et al [12] findings in Japan, Wu et al [13] extended the analysis to multilingual contexts and assessed ChatGPT’s performance on nursing licensure examinations in the United States and China, identifying a significant linguistic bias in multilingual processing, with English inputs yielding substantially higher accuracy than Chinese inputs. This concern was further amplified by Zong et al [11], whose research indicated that ChatGPT (OpenAI) failed to pass the CNNLE, potentially due to limitations in linguistic data, insufficient comprehension of cultural and policy nuances, and weaknesses in numerical computation capabilities. Consequently, for Chinese text, there is an urgent need for a high-performing Chinese language model capable of matching the capabilities of English models in language understanding, cultural insight, and specialized knowledge, in order to bridge the performance disparities due to linguistic and cultural mismatches, as well as the technological limitations in multilingual environments. To this end, this study aims to evaluate the performance of DeepSeek and other leading LLMs on the CNNLE, focusing on accuracy, reasoning depth, and robustness.

Among the LLMs evaluated, DeepSeek, a recently prominent open-source LLM, has demonstrated competitive advantages over other mainstream LLMs in Chinese text generation, question answering, and reading comprehension tasks, achieved through in-depth training on extensive Chinese and English datasets [14-17]. However, current research concerning DeepSeek’s application within the CNNLE remains limited. Therefore, this study comprehensively investigates its potential application to provide crucial references for future medical language model development. In addition, this study conducted a comparative performance evaluation of leading LLMs such as ChatGPT and Gemini (Google) and performed an in-depth comparative analysis of their knowledge comprehension depth and clinical reasoning abilities within the Chinese medical linguistic context. This evaluation sought to comprehensively assess the robustness of each LLM and their confidence in addressing professional inquiries, ultimately providing empirical evidence to optimize language model architectures tailored for Chinese medical scenarios and to facilitate the advancement of cross-lingual medical education technology innovation.

## Methods

### Overview

This section outlines the comprehensive methodology used to evaluate the performance of the 4 selected LLMs on the 2024 CNNLE. We describe the study design, data source, LLM selection criteria, and the specific metrics used to measure accuracy, repeatability, confidence, and robustness.

### Study Design

**Figure 1.**
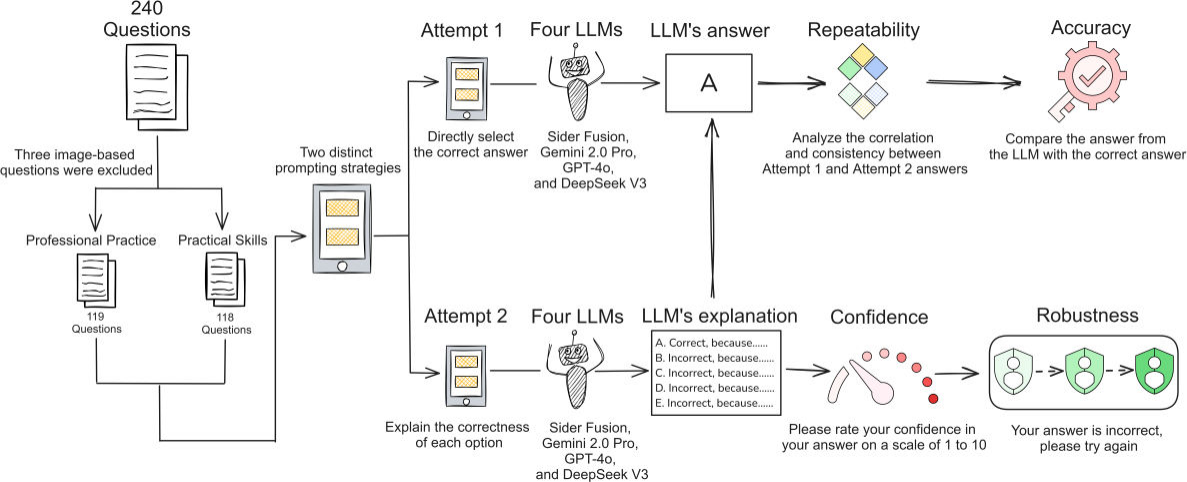
Flowchart of study protocol. LLM: large language model.

This study aimed to evaluate the feasibility of 4 LLMs in answering questions from the CNNLE. Figure 1 illustrates the workflow of this study. First, the 2024 CNNLE questions were input into the LLMs, and their responses were obtained in March 2025 using 2 distinct prompting strategies: (1) Attempt 1, where LLMs were directly instructed to select the correct answer from the provided options and (2) Attempt 2, where LLMs were required to provide a detailed explanation for the correctness of each option before making a selection. Subsequently, the correlation between answers obtained under the different prompting strategies was assessed, and the LLMs’ selected answers were compared against the standard answers to measure their repeatability and accuracy. Building upon Attempt 2, LLMs were further prompted to self-assess their confidence in their answer judgments on a scale of 1 to 10. In addition, an adversarial prompting method was used, involving 3 consecutive challenges to the LLM’s chosen answer within the same session, to evaluate the LLMs’ robustness when responding to professional questions.

### Data Source

The 2024 CNNLE questions were sourced from Baidu Wenku [18]. The CNNLE examination comprises 2 sections: Professional Practice and Practical Skills. The Professional Practice section focuses on assessing the clinical application of medical knowledge related to health and disease, fundamental nursing knowledge and skills, and nursing humanities. The Practical Skills section emphasizes the evaluation of clinical applications such as disease manifestations, treatment principles, health assessment, nursing procedures, nursing techniques, and health education. Each section contains 120 multiple-choice questions (MCQs). Question types include Type A1 (single sentence stem, requiring a single best answer), Type A2 (case summary stem, assessing case comprehension and judgment), Type A3 (case group stem, involving case scenarios and related questions), and Type A4 (serial case stem, providing progressive patient information to gauge understanding of changing conditions and responses). The proportion of each question type may vary by year. For analytical consistency, Types A3 and A4, due to their structural similarities, were merged into a single category (A3/A4).

As image analysis requires external plug-ins, 3 image-based questions (1 from Professional Practice and 2 from Practical Skills) were excluded from the study. To control for potential biases arising from prior interactions, each question was presented to the 4 LLMs within a separate, new chat session during the evaluation. For objectivity and reproducibility, we recorded each LLM’s first complete response verbatim for every question in both Attempt 1 and Attempt 2, without any follow-up prompts, clarifications, or researcher interventions intended to assist the LLMs.

### Measurements

#### LLM Selection

We selected 4 LLMs for this study—DeepSeek V3, GPT-4o (OpenAI), Gemini 2.0 Pro (Google), and Sider Fusion (Vidline Inc)—to balance three key criteria: (1) state-of-the-art performance, (2) diverse technological approaches, and (3) relevance to the Chinese context. These LLMs were chosen not as an exhaustive list, but as a strategic sample representing different archetypes in the current LLM ecosystem. The selected LLMs and their respective archetypes are as follows:

1. DeepSeek V3, introduced by a deep learning technology company in July 2024, was selected for its explicit relevance to the Chinese context. It possesses efficient information retrieval and data analysis capabilities, achieved through deep optimization on Chinese datasets, making it a critical local benchmark for performance on the CNNLE.
2. GPT-4o, released by OpenAI in May 2024, was chosen as the global state-of-the-art benchmark. As an optimized version of GPT-4, it significantly enhances multimodal interaction capabilities and demonstrates outstanding performance in areas such as language understanding, making its performance in the Chinese context a key point of investigation.
3. Gemini 2.0 Pro, launched by Google DeepMind in February 2025, was included to ensure technological diversity. As an advanced model designed to optimize multimodal processing, it represents a major and architecturally distinct competitor to GPT-4o, exhibiting exceptional learning and reasoning abilities in complex cross-lingual and cross-domain tasks.
4. Sider Fusion was selected to represent a metamodel or aggregator approach. As the core function of the Sider intelligent browser extension, it integrates and intelligently selects from official application programming interfaces of various mainstream LLMs, such as GPT-4o mini, Claude 3 Haiku, Gemini 1.5 Flash, and Llama 3. Including Sider Fusion allows us to evaluate the performance of a proxy that simulates a common real-world user experience, where the underlying model is dynamically chosen, rather than assessing a single, static model.

#### Accuracy and Repeatability

Attempt 1 was designed to measure the LLM’s intuitive knowledge matching ability for structured questions, requiring it to directly identify the optimal answer from the options. Attempt 2 aimed to evaluate the LLM’s comprehension and reasoning depth, requiring the LLM to explain why each option was correct or incorrect before providing the final answer. By calculating the answer correlation between the 2 strategies and comparing the LLM’s responses against the standard answers, the accuracy, repeatability, and internal consistency of the LLM’s reasoning logic in applying professional knowledge could be quantified.

#### Confidence and Robustness

During Attempt 2, which required LLMs to explain each option before selection, this study introduced a confidence prompt (eg, “Please rate your confidence in the answer on a scale of 1‐10”) to analyze the LLM’s confidence level regarding its responses. Concurrently, after each answer selection, the study incorporated an adversarial prompt (eg, “Your answer is incorrect, please try again”), followed by 3 rounds of systematic validation within the same session. This aimed to comprehensively examine the LLM’s cognitive resilience and knowledge consistency when faced with persistent challenges.

### Statistical Analysis

All statistical analyses were performed using SPSS (version 26.0; IBM Corp). A *P* value <.05 was considered statistically significant. The Pearson chi-square test was used to compare differences in results among the different LLMs. The answer correlation between Attempt 1 and Attempt 2 was calculated to determine the replicate reliability of each LLM; a Pearson correlation coefficient (*r*)≥0.7 was considered indicative of good replicate reliability. UpSet plots and the heatmap were generated by using the “BioLadder” plotting website [19]. Furthermore, referencing the study by Krishna et al [20], the following thresholds were established for this study:

1. An LLM was considered to have poor repeatability if the consistency of answer choices between Attempt 1 and Attempt 2 (percentage of identical answers selected) was below 90%.
2. An LLM was deemed to have poor robustness if its answer changed in more than 10% of cases following the first round of adversarial questioning.
3. A confidence score of 8 or higher was defined as high confidence, while a score below 8 was defined as low confidence.
4. High confidence scores for incorrect answers were considered overconfidence. An LLM was classified as frequently overconfident if the incidence of overconfidence (ie, the proportion of incorrect answers with high confidence scores) exceeded 10%.

### Ethical Considerations

The data for this study consisted of publicly available multiple-choice questions from the 2024 Chinese National Nurse Licensure Examination, which were sourced from a publicly accessible online repository as described in the Methods section. As this study did not involve human participants, the recruitment of subjects and the collection of personal data were not applicable. Therefore, the requirement for written informed consent was waived. All procedures were approved by the Ethics Committee of the First Affiliated Hospital of Shandong First Medical University (approval number YXLL-KY-2025‐031). We confirm that all data were handled in a manner that respected the intellectual property of the source. No compensation was provided to any party for the data used in this study.

## Results

### Overview

This section presents the empirical findings from our comparative evaluation of the 4 LLMs. The results are organized by the primary performance dimensions assessed: accuracy across different question types and exam sections, repeatability between prompting strategies, confidence calibration, and robustness under adversarial conditions.

### Accuracy

The accuracy rates of the 4 LLMs were evaluated through 2 independent attempts, with the results presented in Table 1. Overall, Gemini 2.0 Pro and DeepSeek V3 demonstrated significant advantages, with overall accuracy rates exceeding 83% in both attempts. Specifically, DeepSeek V3 achieved the highest accuracy in the first attempt at 88.19% (209/237), while Gemini 2.0 Pro’s accuracy improved further in the second attempt to 86.50% (205/237), surpassing the accuracy of DeepSeek V3 in the second attempt (*P*<.001). In contrast, the performance of GPT-4o (Attempt 1: 161/237, 67.93%; Attempt 2: 166/237, 70.04%) and Sider Fusion (Attempt 1: 153/237, 64.56%; Attempt 2: 151/237, 63.71%) was comparatively lower (all *P*<.001).

**Table 1.** Accuracy rates of the 4 large language models on the Chinese National Nurse Licensure Examination multiple-choice questions.

Categories		DeepSeek V3	GPT-4o	Gemini 2.0 Pro	Sider Fusion
Attempt 1, n (%)					
Professional practice (n=119)		105 (88.23)	78 (65.55)	98 (82.35)	69 (57.98)
Practical skills (n=118)		104 (88.14)	83 (70.34)	101 (85.59)	84 (71.19)
Overall (N=237)		209 (88.19)	161 (67.93)	199 (83.97)	153 (64.56)
Attempt 2, n (%)					
Professional practice (n=119)		96 (80.67)	80 (67.23)	102 (85.71)	72 (60.50)
Practical skills (n=118)		106 (89.83)	86 (72.88)	103 (87.29)	79 (66.95)
Overall (N=237)		202 (85.23)	166 (70.04)	205 (86.50)	151 (63.71)

Notably, most LLMs tended to perform better on the Practical Skills section compared to the Professional Practice section. However, significant intragroup differences between the 2 sections were observed only for Sider Fusion and DeepSeek V3: in the first attempt, Sider Fusion’s accuracy in Professional Practice (69/119, 57.98%) was 13.21 percentage points lower than in Practical Skills (84/118, 71.19%; *P*=.03); in the second attempt, DeepSeek V3’s accuracy in Professional Practice (96/119, 80.67%) showed a statistically significant difference of 9.16 percentage points compared to Practical Skills (106/118, 89.83%; *P*=.047). For the remaining LLMs, the differences in performance between the 2 sections did not reach statistical significance (minimum *P*=.34).

In the analysis of question-type specificity, the performance of each LLM on Type A1 questions across the 2 attempts was as follows: DeepSeek V3 scored 90.30% (149/165) and 84.85% (140/165), GPT-4o scored 69.09% (114/165) and 68.48% (113/165), Gemini 2.0 Pro scored 86.06% (142/165) and 87.88% (145/165), and Sider Fusion scored 65.45% (108/165) and 63.64% (105/165), respectively. For Type A2 questions, the accuracy rates in the 2 attempts were as follows: DeepSeek V3 scored 82.61% (57/69) and 85.51% (59/69), GPT-4o scored 63.77% (44/69) and 72.46% (50/69), Gemini 2.0 Pro scored 78.26% (54/69) and 82.61% (57/69), and Sider Fusion scored 60.87% (42/69) and 62.32% (43/69), respectively. All LLMs achieved 100% accuracy (3/3) on Type A3/A4 questions. Furthermore, statistical analysis indicated that there were no significant intragroup differences in performance across the different question types (A1, A2, A3/A4) for any LLM (minimum *P*=.10), suggesting that question type had minimal impact on LLM accuracy.

**Figure 2.**
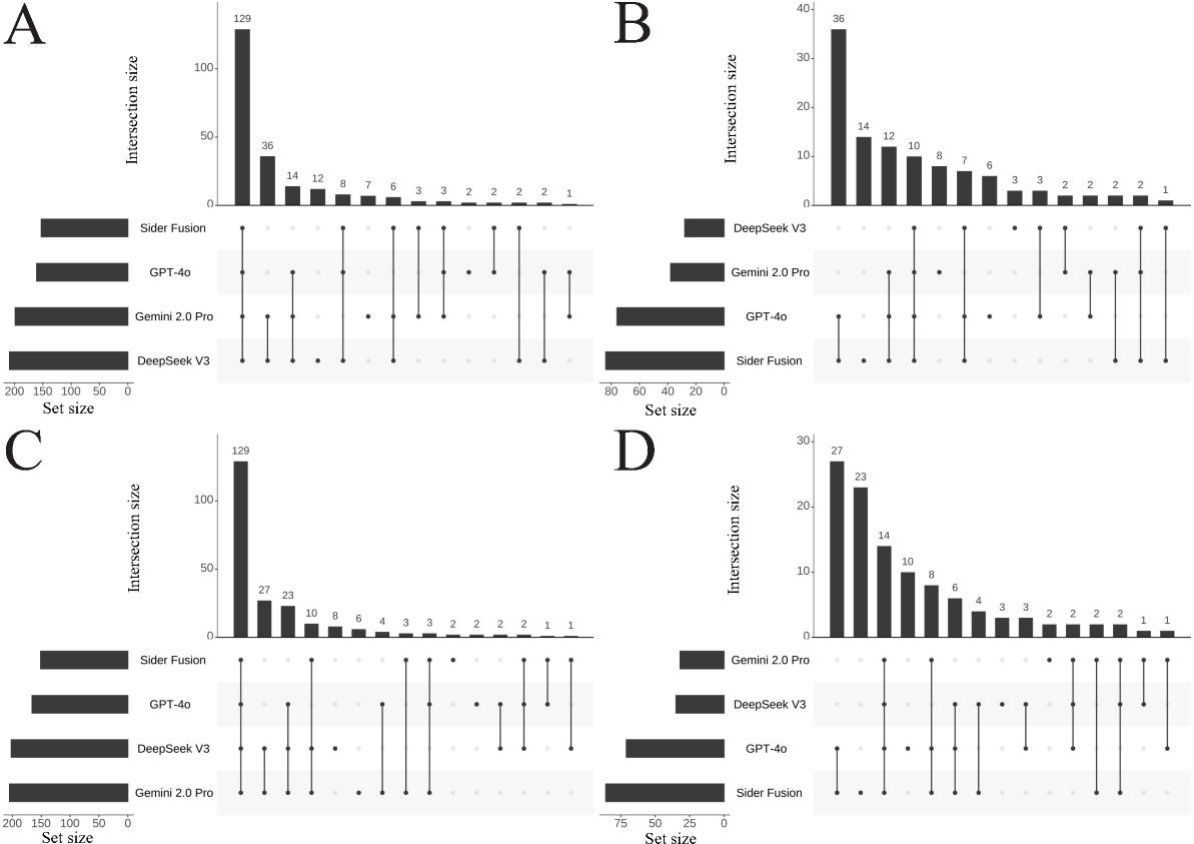
UpSet plots showing the intersection of correct (**A **and** C**) and incorrect (**B and D**) answers among the different LLMs for all CNNLE multiple-choice questions in Attempt 1 (**A and B**) and Attempt 2 (**C and D**). The horizontal bars on the left, labeled set size, represent the total count of items belonging to each respective LLM. The dot matrix below the main plot defines the specific combinations of LLMs being considered, where connected dots indicate which LLMs are part of an intersection. The vertical bars above this matrix then quantify the size (ie, the number of questions) for each of these specific intersections. CNNLE: Chinese National Nurse Licensure Examination; LLM: large language model.

Figure 2 illustrates the intersection distribution of correct and incorrect answers for all CNNLE MCQs across the LLMs in Attempt 1 and Attempt 2. DeepSeek V3 possessed the highest number of unique correct answers in both attempts (12 and 8, respectively), demonstrating a significant advantage in identifying unique correct answers. This was followed by Gemini 2.0 Pro (7 and 6 unique correct answers, respectively) and the third GPT-4o (2 unique correct answers in both attempts). Conversely, Sider Fusion dominated the distribution of unique incorrect answers, with 14 and 23 unique errors in the respective attempts. Notably, the 4 LLMs showed high stability regarding consensus on correct answers: the intersection of correct answers for all MCQs remained stable at 129 items (Figure 2A and C), whereas the consensus on incorrect answers was 10 and 14 items, respectively (Figure 2B and D).

**Figure 3.**
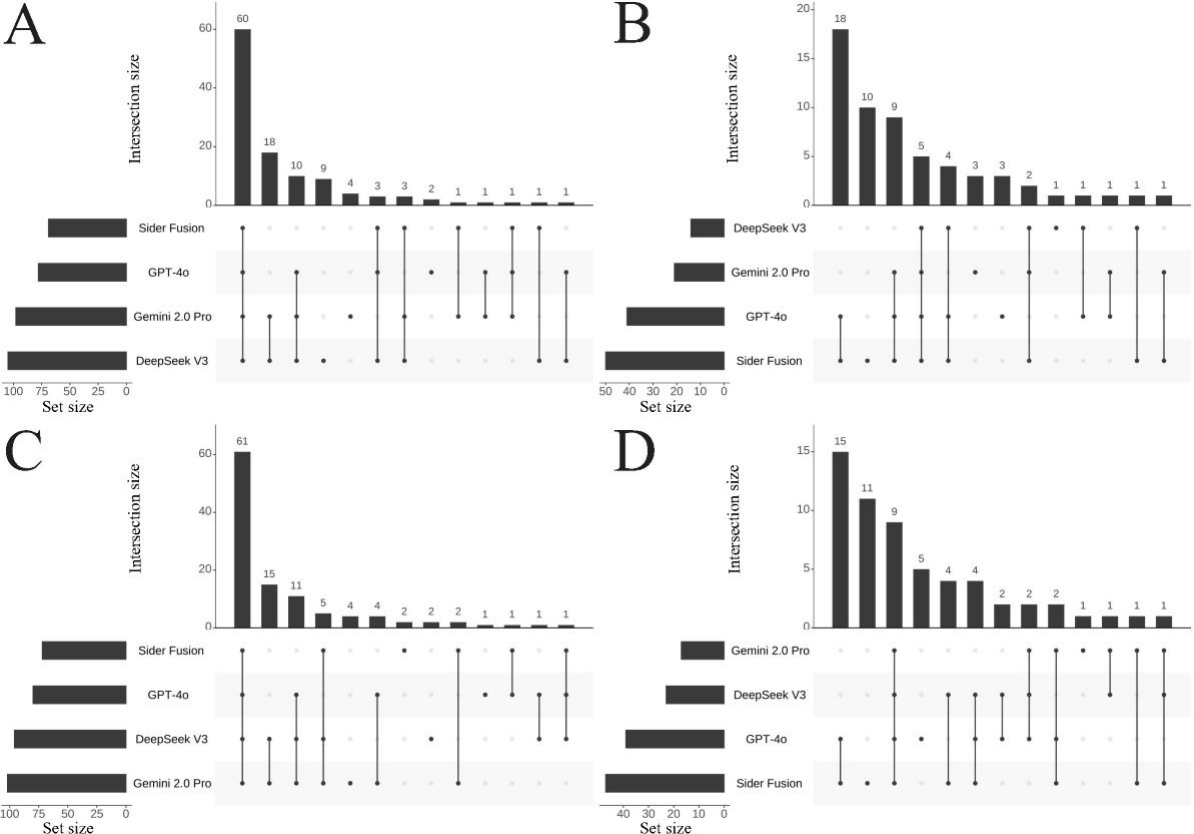
UpSet plots showing the intersection of correct (**A and C**) and incorrect (**B and D**) answers among the different LLMs for CNNLE Professional Practice multiple-choice questions in Attempt 1 (**A and B**) and Attempt 2 (**C and D**). The horizontal bars on the left, labeled set size, represent the total count of items belonging to each respective LLM. The dot matrix below the main plot defines the specific combinations of LLMs being considered, where connected dots indicate which LLMs are part of an intersection. The vertical bars above this matrix then quantify the size (ie, the number of questions) for each of these specific intersections. CNNLE: Chinese National Nurse Licensure Examination; LLM: large language model.

**Figure 4.**
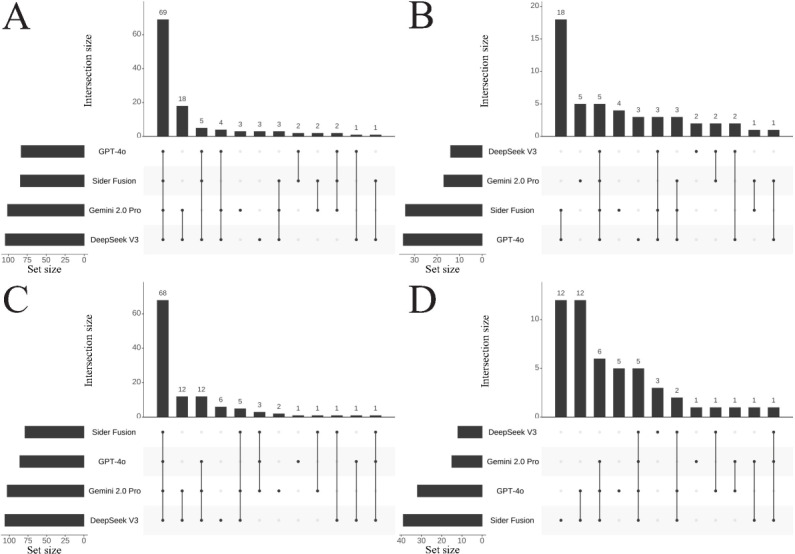
UpSet plots showing the intersection of correct (A and C) and incorrect (B and D) answers among the different LLMs for CNNLE Practical Skills multiple-choice questions in Attempt 1 (**A and B**) and Attempt 2 (**C and D**). The horizontal bars on the left, labeled set size, represent the total count of items belonging to each respective LLM. The dot matrix below the main plot defines the specific combinations of LLMs being considered, where connected dots indicate which LLMs are part of an intersection. The vertical bars above this matrix then quantify the size (ie, the number of questions) for each of these specific intersections. CNNLE: Chinese National Nurse Licensure Examination; LLM: large language model.

Similarly, Figure 3 presents the intersection distributions for the CNNLE Professional Practice questions, and Figure 4 presents those for the Practical Skills questions. Key findings include the following: (1) In the Professional Practice section, DeepSeek V3 had 9 and 2 unique correct answers in Attempts 1 and 2, respectively, and only 1 unique incorrect answer appeared in Attempt 1. Gemini 2.0 Pro achieved 4 unique correct answers in both Attempt 1 and Attempt 2, accompanied by 3 and 1 unique incorrect answers, respectively. GPT-4o yielded 2 unique correct answers in Attempt 1 and 1 in Attempt 2, alongside 3 and 5 unique incorrect answers, respectively. Sider Fusion produced no unique correct answers in Attempt 1 but 2 in Attempt 2, while registering 10 and 11 unique incorrect answers in Attempts 1 and 2, respectively. (2) In the Practical Skills section, DeepSeek V3 showed an increasing trend for both unique correct answers (3 and 6) and unique incorrect answers (2 and 3) between Attempt 1 and Attempt 2. In contrast, Sider Fusion completely lacked unique correct answers in this section across both attempts, and its unique incorrect answers surged from 4 in Attempt 1 to 12 in Attempt 2, suggesting potential deficiencies in Sider Fusion’s understanding of clinical operational protocols.

Overall, Figures2^4^ exhibited typical long-tail distributions. Interestingly, a mirror symmetry in count emerged between the correct answers shared exclusively by the Gemini 2.0 Pro and DeepSeek V3 pair and the incorrect answers shared by the GPT-4o and Sider Fusion pair. Specifically, across all MCQs, the number of answers correctly answered only by both Gemini 2.0 Pro and DeepSeek V3 was 36 and 27 in Attempts 1 and 2, respectively; this count mirrored the number of answers incorrectly answered by both GPT-4o and Sider Fusion, which was also 36 and 27 for the respective attempts. In the Professional Practice section, the number of answers correct only for both Gemini 2.0 Pro and DeepSeek V3 was 18 and 15, respectively, again matching the number of incorrect answers shared between GPT-4o and Sider Fusion in that section (18 and 15, respectively). A similar symmetrical distribution was observed in the Practical Skills section.

### Repeatability

Comparison of answer correlation between Attempt 1 and Attempt 2 revealed that all LLMs achieved statistically significant correlation (all *P*<.001). Among them, Gemini 2.0 Pro (*r*=0.82) and DeepSeek V3 (*r*=0.80) demonstrated stronger replicate reliability, outperforming Sider Fusion (*r*=0.61) and GPT-4o (*r*=0.64).

Analysis of answer consistency (percentage of identical answers) between Attempt 1 and Attempt 2 showed that none of the LLMs met the 90% threshold, indicating poor repeatability across the LLMs. The specific data exhibited significant stratification (all *P*<.001): DeepSeek V3 ranked highest with 86.92% (206/237) consistency, maintaining a 1.69% performance margin over the second-ranked Gemini 2.0 Pro (202/237, 85.23%). The third-ranked Sider Fusion (169/237, 71.31%), although slightly ahead of GPT-4o (166/237, 70.04%) by 1.27%, lagged behind the leading LLMs by 15.61 percentage points in consistency.

Among the consistently answered responses (identical answers in both attempts), the proportions of correct responses were DeepSeek V3 (192/237, 81.01%), Gemini 2.0 Pro (187/237, 78.90%), GPT-4o (142/237, 59.92%), and Sider Fusion (126/237, 53.16%). Furthermore, although preliminary data suggested that consistency in correct answers might be higher for Type A1 questions than for Type A2 questions for each LLM, statistical analysis did not confirm this difference as significant: DeepSeek V3 (A1 vs A2: 134/165, 81.21% vs 55/69, 79.71%; *P*=.79), Gemini 2.0 Pro (A1 vs A2: 133/165, 80.61% vs 51/69, 73.91%; *P*=.26), GPT-4o (A1 vs A2: 100/165, 60.61% vs 39/69, 56.52%; *P*=.56), and Sider Fusion (A1 vs A2: 88/165, 53.33% vs 35/69, 50.72%; *P*=.72).

### Confidence

All LLMs demonstrated a significant tendency toward high confidence (score ≥8) in the majority of their responses: Sider Fusion (222/237, 93.67%), GPT-4o (232/237, 97.89%), Gemini 2.0 Pro (234/237, 98.73%), and DeepSeek V3 (232/237, 97.89%). Further analysis of incorrect answers revealed a prevalent phenomenon of overconfidence among the LLMs: the proportion of high confidence among incorrect answers reached 88.37% (76/86) for Sider Fusion, 94.37% (67/71) for GPT-4o, 93.75% (30/32) for Gemini 2.0 Pro, and 94.29% (33/35) for DeepSeek V3. Moreover, except for DeepSeek V3, where no statistically significant difference was found between confidence level and answer accuracy (high confidence: 199/202, 98.51% correct vs 33/35, 94.29% incorrect; *P*=.16), statistically significant differences between confidence level and answer accuracy were observed for the other LLMs: Sider Fusion (high confidence: 146/151, 96.69% correct vs 76/86, 88.37% incorrect; *P*=.01), GPT-4o (high confidence: 165/166, 99.40% correct vs 67/71, 94.37% incorrect; *P*=.03), and Gemini 2.0 Pro (high confidence: correct 204/205, 99.51% vs 30/32, 93.75% incorrect; *P*=.049).

### Robustness

In the first round of adversarial questioning, Sider Fusion had a response change rate of 33.76% (80/237), followed by GPT-4o (85/237, 35.86%), DeepSeek V3 (78/237, 32.91%), and Gemini 2.0 Pro (75/237, 31.65%). The differences in change rates among the 4 LLMs were statistically significant (all *P*<.001). Furthermore, the quality of answers maintained (ie, not changed) during adversarial questioning showed significant stratification among the LLMs (all *P*<.001): Gemini 2.0 Pro led with the highest stable accuracy rate (accuracy of unchanged answers) at 91.98% (149/162), closely followed by DeepSeek V3 at 91.19% (145/159). GPT-4o and Sider Fusion ranked third and fourth, with stable accuracy rates of 78.95% (120/152) and 73.25% (115/157), respectively.

After 3 rounds of adversarial questioning, the final accuracy rates were 59.07% (140/237) for Sider Fusion, 60.34% (143/237) for GPT-4o, 72.15% (171/237) for DeepSeek V3, and 72.57% (172/237) for Gemini 2.0 Pro. During these 3 rounds, Sider Fusion incorrectly changed 27 initially correct answers to incorrect ones, while DeepSeek V3 changed 38, GPT-4o changed 41, and Gemini 2.0 Pro changed 43. Regarding error correction capability (changing an initially incorrect answer to the correct one after challenge), Sider Fusion successfully corrected 25 incorrect answers, GPT-4o corrected 18, and DeepSeek V3 corrected 17. However, Gemini 2.0 Pro only successfully corrected 10 incorrect answers.

## Discussion

### Principal Findings

**Figure 5.**
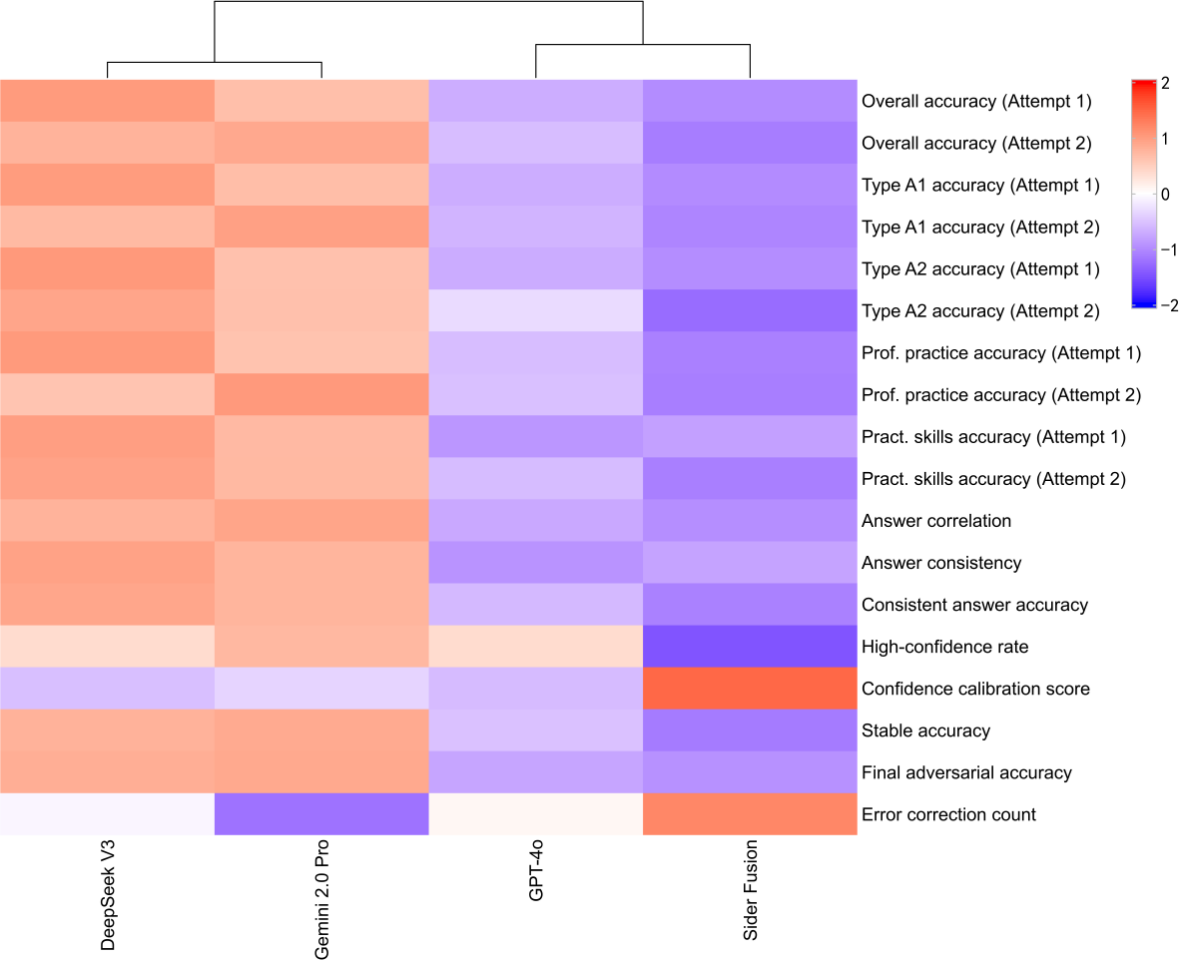
Heatmap summarizing the comparative performance of the 4 LLMs across key evaluation metrics. In this heatmap, cell colors represent normalized performance scores for each metric, with warmer colors (red and orange) indicating stronger performance and cooler colors (blue and purple) indicating weaker performance. LLM: large language model; Pract.: practical; Prof.: professional.

This study systematically evaluated the performance of DeepSeek V3, Gemini 2.0 Pro, GPT-4o, and Sider Fusion on the CNNLE, finding significant differences in overall performance among the LLMs, as summarized in the heatmap in Figure 5. Some LLMs demonstrated distinct advantages, potentially owing to their deep optimization for the Chinese context and cross-modal training mechanisms. However, all LLMs exhibited a risk of hallucination, generating outputs that appear plausible but lack factual accuracy. This finding implies that for educators, developers, and policymakers alike, the focus must shift from merely pursuing correct LLM answers to building an ecosystem that can safely manage the LLMs’ inherent potential for error.

Furthermore, none of the LLMs achieved the desired 90% threshold for answer consistency, indicating that even the best-performing LLMs exhibit repeatability deficits. This finding corroborates previous research by Krishna et al [20], Wang et al [21], and Kochanek et al [22]. Potential underlying reasons may include the stochasticity of model parameters, biases in fine-tuning data, sensitivity to prompt design, and inherent limitations in the LLMs’ comprehension and reasoning abilities regarding complex, specialized knowledge. This inherent inconsistency poses a significant challenge to the application of LLMs, particularly in highly standardized educational scenarios where it could undermine fairness in assessment, such as by providing different evaluations for similar answers in automated grading. Addressing this deficit at a technical level is therefore crucial. For future LLM design, we recommend focusing on two key aspects: First, refining and dynamically optimizing decoding strategies, such as contextually adapting temperature and top-p parameters, to enhance answer consistency while maintaining plausibility. Second, incorporating task-oriented consistency reward functions and reinforcement learning environments during training to positively incentivize the generation of more repeatable and reliable answers.

This study observed a mirror symmetry phenomenon where the set of correct answers shared between the Gemini 2.0 Pro and DeepSeek V3 pair mirrored the set of incorrect answers shared between the GPT-4o and Sider Fusion pair. This reveals intrinsic differences in knowledge acquisition and reasoning mechanisms among the LLM cohort: simple questions were generally answered correctly, while difficult ones were commonly missed, and questions with high discriminatory power amplified the performance disparities between LLMs [23-25]. These performance disparities likely stem from underlying differences in model architecture and training strategies. DeepSeek V3, leveraging its mixture-of-experts architecture and knowledge distillation techniques, can achieve specialized processing for different data types [26]. Although the architecture of Gemini 2.0 Pro is not publicly disclosed, we speculate that its training strategy similarly focuses on efficient indexing and retrieval of domain-specific knowledge [27]. Conversely, GPT-4o and Sider Fusion might prioritize exploring complex reasoning paths. However, in complex situations with insufficient domain knowledge or ambiguous semantic information, this strategy becomes susceptible to interference, ultimately leading to a consensus on incorrect answers [23,28]. Therefore, LLM errors are not merely random noise but are manifestations of systematic biases in specific domain knowledge or reasoning strategies [29-31]. Intriguingly, these systematic biases might bear a striking resemblance to human expert error patterns in certain complex tasks. As demonstrated by Bernstein et al [32], no significant differences were found between GPT-3.5 and human answers regarding the presence of incorrect information (*P*=.58), likelihood of causing harm (*P*=.35), extent of possible harm (*P*=.84), and agreement with perceived consensus in the medical community (*P*=.35). Similarly, research by Cabral et al [33] found that although GPT-4 might exhibit a higher error frequency than resident physicians in clinical reasoning (13.8% vs 2.8%; *P*=.04), its error frequency did not differ significantly from that of experienced attending physicians (13.8% vs 12.5%; *P*=.89). This convergence of error patterns suggests that the safest and most effective model for high-stakes fields such as health care is likely not independent human or AI decision-making, but rather a deeply integrated human-AI collaborative intelligence. Within such a paradigm, educators can design human-AI pairing case studies, training students to supervise, question, and ultimately form synergistic decisions with LLM recommendations, thereby learning to identify and mitigate shared cognitive blind spots. Crucially, this mitigation process rests on a thorough understanding of the biases specific to both humans and LLMs. It is well-established that human cognitive biases can be mitigated to some extent through increased awareness, education, and data-driven decision-making methods [34,35]. In-depth research into the systematic biases of LLMs not only contributes to enhancing the reliability of AI systems but may also, in turn, deepen our understanding of the origins and mechanisms of human cognitive biases. A detailed comparative analysis between LLM systematic biases and human cognitive biases could therefore equip future health care professionals with the critical awareness needed to effectively supervise and collaborate with LLMs, ensuring safer and more reliable outcomes.

Consistent with findings by Krishna et al [20], our study reveals a cautionary phenomenon: Although the LLMs exhibited high confidence in most responses, there was a clear disconnect between their confidence levels and the actual accuracy of their answers, with incorrect answers frequently accompanied by high confidence scores. This LLM overconfidence trap could lead health care professionals to inappropriately trust incorrect recommendations, potentially resulting in diagnostic biases, treatment errors, and other clinical risks, ultimately threatening patient safety [36,37]. Notably, DeepSeek V3 was the only LLM where confidence level and actual accuracy showed no statistically significant difference (*P*=.16). This might be related to its deep optimization on Chinese medical texts during training, enabling the LLM to more accurately capture linguistic nuances and cultural context when parsing CNNLE questions, thereby reducing confidence misjudgments arising from semantic ambiguity or cultural misinterpretation [26]. It is recommended that future LLM updates prioritize confidence calibration as a core optimization goal. This can be pursued by systematically representing medical knowledge and its internal relationships, quantifying the uncertainty of LLM outputs, and developing more discriminating confidence assessment systems. However, ensuring these crucial improvements are implemented consistently requires more than developer self-regulation. This is where policy plays a critical role. From a policymaking perspective, regulatory bodies should consider establishing confidence calibration performance as a mandatory disclosure metric for AI tools in the medical and educational sectors. This would ensure that health care and educational institutions can make informed decisions based on transparent, quantifiable risk data when procuring and deploying these tools, thereby establishing an essential institutional safeguard to protect patients and learners.

The robustness test results in this study present an interesting paradox: while Gemini 2.0 Pro performed best at maintaining its initial answers, its error correction efficacy was relatively limited. Conversely, although Sider Fusion exhibited the highest rate of answer changes, it demonstrated the strongest error correction capability. This finding aligns with the “stability–flexibility trade-off” theory [38], suggesting a potential imbalance in current LLMs between knowledge representation stability and adaptive adjustment capabilities. From a clinical decision support perspective, this imbalance could pose potential risks: on one hand, excessive adherence to incorrect answers (high stability coupled with low correction capability) might lead to the persistence of systematic errors; on the other hand, excessive correction of initially correct answers (high correction capability coupled with low stability) could induce decision volatility. However, for designers of licensure preparation platforms, this paradox is not a deficit but instead offers significant practical guidance. It inspires a multimodal product design that moves beyond a one-size-fits-all approach: developers can offer a tutor mode powered by a high-stability LLM for knowledge consolidation, alongside a sparring partner mode driven by a high-flexibility LLM to stimulate critical thinking. Therefore, we recommend that future LLM development not only focuses on balancing accuracy, robustness, and correction sensitivity at the LLM level, but also strategically considers how to translate these different performance characteristics into diverse pedagogical tools at the product design level, with the ultimate goal of empowering educators to cultivate future health care professionals capable of collaborating safely and effectively with imperfect LLMs and ultimately safeguarding decision-making in real-world medical environments.

### Limitations

This study possesses several limitations. First, our evaluation was constrained in scope. We excluded 3 image-based questions, which limited a full assessment of the LLMs’ multimodal capabilities. Our model selection, while representative, did not include direct and independent evaluations of other prominent LLMs such as Claude 3, LLaMA, or Mistral, restricting the generalizability of our findings. Furthermore, our study’s reliance on MCQs, though beneficial for quantification, does not capture essential practical competencies in nursing, such as procedural skills or patient communication. Future research should incorporate multimodal data, broaden the comparative analysis to include a wider array of LLMs, and explore more holistic appraisal approaches to better understand the potential and constraints of LLMs in nursing education and practice.

Second, our robustness testing methodology, while revealing, was simplistic. The adversarial prompt used represents a blunt, generic negation and does not capture the complexity and nuance of authentic clinical feedback. Consequently, our test may not adequately simulate the multifaceted interference patterns of real-world scenarios, potentially underestimating the LLMs’ vulnerability. Future research should therefore focus on devising more ecologically valid adversarial testing methodologies that move beyond simple negation to probe higher-order reasoning. This could range from presenting contradictory patient data to test dynamic reasoning, to challenging an LLM with a competing differential diagnosis to assess comparative argumentation, and finally, requiring the LLM to explain the rationale behind its recommended treatment plan to evaluate its ability to weigh trade-offs based on clinical guidelines.

Finally, during the second round of the robustness test, we observed that after their answer from the previous round was challenged, some LLMs would occasionally provide an ambiguous response containing 2 answer choices. To maintain procedural consistency, we addressed this phenomenon by reapplying the same standardized adversarial prompt: “Your answer is incorrect, please try again.” This prompt successfully guided the LLMs to provide a single, definitive answer in the third round. Although the responses from the second round were not included in our analysis, we now recognize these occasional ambiguous answers as valuable data on LLM decision-making instability under persistent pressure. Had we quantified these instances as fallback events, we could have conducted a more in-depth analysis of the LLMs’ cognitive resilience and decision confidence boundaries. Therefore, we strongly recommend that future research separately quantify an LLM’s autonomous accuracy on definitive responses and its fallback rate on ambiguous ones to create a more nuanced reliability profile.

### Conclusions

This study evaluated the performance of 4 LLMs on the CNNLE, revealing the relative advantages of DeepSeek V3 and Gemini 2.0 Pro. However, a concerning finding was the LLMs’ tendency for overconfidence, which stood in stark contrast to their limited accuracy. This highlights a fundamental deficit in the cognitive reliability of current LLMs when applied to complex professional reasoning tasks. Such overconfidence is particularly perilous within the safety-critical domain of health care. Furthermore, the “stability-flexibility trade-off” paradox suggests a potential imbalance in current LLMs between the stability of knowledge representation and adaptive adjustment capabilities. The true use of LLMs in nursing education and practice demands more than just correct answers; it crucially depends on the reliability of their reasoning, calibrated confidence, and robustness against real-world complexities. The path forward requires a paradigm shift in LLM development and evaluation, prioritizing genuine cognitive reliability and trustworthiness over merely simulating knowledgeable responses.

## Abbreviations

AI: artificial intelligence
CNNLE: Chinese National Nurse Licensure Examination
LLM: large language model
MCQ: multiple-choice question
